# Defects in mismatch repair: the Achilles heel of estrogen receptor positive breast cancer with intrinsic endocrine therapy resistance?

**DOI:** 10.18632/oncoscience.363

**Published:** 2017-09-21

**Authors:** Svasti Haricharan, Matthew J. Ellis

**Affiliations:** Lester and Sue Smith Breast Center, Baylor College of Medicine, Houston, TX 77030, USA

Estrogen receptor signaling has long been associated with DNA damage repair in subtle and complex ways and ER-induced proliferation appears to downregulate the complex repertoire of DNA repair pathways and downstream effectors in both normal and cancer cells [1]. In the context of normal mammary gland development, this finding is perhaps not surprising, since the breast is characterized by repeated rounds of rapid proliferation and expansion followed by involution in response to reproductive hormones. To achieve such a rapid accumulation of cellular mass, check points must be suspended, perhaps even at the expense of an increased mutational load and cancer risk. Our recent discovery of a role for mismatch repair defects (MMRD) in endocrine therapy resistance, specifically within the MutL pathway, therefore builds on decades of research on links between ER signaling and DNA damage repair. Our work exposes a very specific link between mismatch repair deficiency involving genes serving the MutL complex and estrogen receptor independent proliferation [2].

Uncovering the role of MutL in intrinsic endocrine therapy resistance was made possible by the generation of somatic mutation and gene expression profiles from ER+ breast cancers accrued from patients treated with neoadjuvant endocrine therapy [3,4]. These clinical trial samples were critical because the significance of the MMRD was missed in earlier ‘omic studies of breast cancer because hitherto analyses involved cases were not specifically annotated for response to endocrine drugs. Furthermore, the type of MMRD defects present in ER+ breast cancers often do not demonstrate the extreme rates of hypermutation and microsatellite instability seen in classic MMRD-driven cancers that arise in the colon and endometrium and as a result the role of MMRD in breast cancer has been underappreciated [5]. Indeed, a comparison of the mutational profiles associated with MutL gene defects in ER+ breast cancer vs other cancer types suggests a predominance of missense alleles rather than the microsatellite instability (MSI) signatures associated with complete loss of gene function. A plausible hypothesis is, therefore, that somatic mutations and loss of expression in MutL genes observed in ER+ breast cancer are sufficient to disrupt the ability of the MutL complex to signal to checkpoint regulators like Chk2, producing slippage of the otherwise tight link between ER and CDK4/6, while not disrupting DNA repair to the point of full-blown hypermutation and MSI. This obviously creates a diagnostic problem, as standard diagnostics for MMRD and MSI are not sufficiently sensitive in the context of ER+ breast cancer. While occasional fully evolved MMRD ER+ breast cancers are beginning to be described [6], we argue that standard tests for MMRD, particularly MSI, reveal only the very tip of the breast cancer MMRD iceberg.

The question of accurately diagnosing MMRD in breast cancer is critical for several developing therapeutic hypotheses. First, the sensitivity of MutL defective ER+ breast cancer to CDK4/6 inhibitors (palbociclib, abameciclib or ribociclib) both in experimental model systems and in human tumors suggests we may be able to target adjuvant CDK4/6 therapy more effectively. For many patients, endocrine therapy is effective and provides life-long protection from relapse. However, for patients with a MutL defective ER+ tumor, the tumor remains in a proliferative state despite endocrine therapy and therefore relapse is more common. Here a CDK4/6 inhibitor would be expected to have a much larger impact on relapse due to its ability to achieve cell cycle control because defective CHK2-mediated CDK4/6 inhibition leads to increased sensitivity to CDK4/6 inhibition. Interestingly CDK4/6 inhibitors may also alter the immune microenvironment and promote an antitumor immune response [7]. Ultimately the increased immunogenicity associated with MMR-loss and high mutation load may be the “Achilles heel” of high-risk ER+ breast cancer. The recent report of responses of ER+ primary breast cancers to combinations of the PD1 inhibitor, palbociclib and chemotherapy is noteworthy in this regard and provokes the question of whether palbociclib and PD1 inhibition are both targeting a population of MutL/MMRD defective tumors for whom the current standard of care of cytotoxic chemotherapy and long term endocrine therapy is too often ineffective [8].

**Figure 1:**
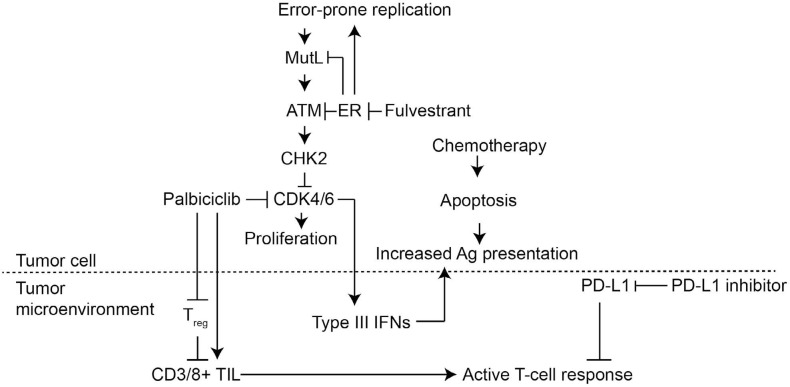
Working model for therapeutic combinations that may prove effective in treating ER+ breast cancer patients with MutL-defects The MutL subset of mismatch repair is inhibited by ER signaling, which is partially responsible for the error-prone proliferation associated with estrogen stimulation in the breast. When ER signaling is inhibited by endocrine therapy, a breast cancer cell with competent MutL activates Chk2 in response to the accumulated DNA damage, which in turn arrests the cell cycle. In a MutL-deficient cell, on the other hand, Chk2 activation is muted and the cell cycle continues unchecked. Therefore, administration of a CDK4/6 inhibitor to arrest cell cycle downstream of Chk2 is effective in stopping cell proliferation. Additionally, CDK4/6 inhibitors may also alter the tumor microenvironment by inhibiting proliferation of regulatory T-cells (T_reg_). This increases the number of tumor-infiltrating lymphocytes (TILs), thereby enabling a stronger cytotoxic T-cell response. In parallel, CDK4/6 inhibition also stimulates type III interferon (IFN) signaling in the tumor cells, thereby increasing antigen presentation by the tumor cell. Chemotherapy which induces apoptosis and release of antigens can also increase antigen presentation to the immune system. In both these cases, it is possible that because tumor cells with MutL-defects have more mutated proteins, the antigens they present will be more immunogenic. A PD-1 inhibitor may be helpful in this setting by reducing apoptosis in antigen specific CD8+ killer T-cells while increasing apoptosis in CD4+ T_reg_ cells.

## References

[1] Caldon C.E. (2014). Frontiers in Oncology.

[2] Haricharan S. (2017). Cancer Discov.

[3] Ellis M.J. et al J Clin Oncol.

[4] Ma C.X. (2017). et al Clin Cancer Res.

[5] Alexandrov L.B. Nature.

[6] Davies H.M. (2017). et al Cancer Research.

[7] Goel S Nature.

[8] Nanda R (2017). Journal of Clinical Oncology.

